# Species distribution modeling and molecular markers suggest longitudinal range shifts and cryptic northern refugia of the typical calcareous grassland species *Hippocrepis comosa* (horseshoe vetch)

**DOI:** 10.1002/ece3.2811

**Published:** 2017-02-21

**Authors:** Martin Leipold, Simone Tausch, Peter Poschlod, Christoph Reisch

**Affiliations:** ^1^ Institute of Plant SciencesUniversity of RegensburgRegensburgGermany

**Keywords:** AFLP, genetic structure, grassland, phylogeography

## Abstract

Calcareous grasslands belong to the most diverse, endangered habitats in Europe, but there is still insufficient information about the origin of the plant species related to these grasslands. In order to illuminate this question, we chose for our study the representative grassland species *Hippocrepis comosa* (Horseshoe vetch). Based on species distribution modeling and molecular markers, we identified the glacial refugia and the postglacial migration routes of the species to Central Europe. We clearly demonstrate that *H. comosa* followed a latitudinal and due to its oceanity also a longitudinal gradient during the last glacial maximum (LGM), restricting the species to southern refugia situated on the Peninsulas of Iberia, the Balkans, and Italy during the last glaciation. However, we also found evidence for cryptic northern refugia in the UK, the Alps, and Central Germany. Both species distribution modeling and molecular markers underline that refugia of temperate, oceanic species such as *H. comosa* must not be exclusively located in southern but also in western of parts of Europe. The analysis showed a distinct separation of the southern refugia into a western cluster embracing Iberia and an eastern group including the Balkans and Italy, which determined the postglacial recolonization of Central Europe. At the end of the LGM,* H. comosa* seems to have expanded from the Iberian refugium, to Central and Northern Europe, including the UK, Belgium, and Germany.

## 1 Introduction

Calcareous grasslands are among the most species‐rich ecosystems in Central Europe (Korneck, Schnittler, & Klingenstein, 1998; Sadlo, Chytry, & Pysek, 2007; WallisDeVries, Poschlod, & Willems, 2002), and many studies have been conducted to understand the ecological mechanisms behind this observation (Cornish, 1954; Dutoit & Alard, 1995; Gibson & Brown, 1991; Kahmen, Poschlod, & Schreiber, 2002; Poschlod, Kiefer, Trankle, Fischer, & Bonn, 1998; Römermann, Bernhardt‐Römermann, Kleyer, & Poschlod, 2009). Notwithstanding these efforts, there is still a major gap about the origin of the plant species associated with this habitat type.

Calcareous grasslands are regarded as seminatural landscapes (Karlik & Poschlod, 2009), created and maintained by human activities—particularly forest clearing and subsequent grazing with livestock (Kahmen & Poschlod, 2004; Poschlod & WallisDeVries, 2002; Schmidt, Fischer, & Becker, 2007). However, they may also already have existed in the periglacial zone in Central Europe during the glacial period (Kunes, Pelankova, & Chytry, 2008; Pokorný, 2005). Recent studies even postulated the occurrence of temperate tree species in so‐called cryptic refugia probably till 45°N latitude (Kunes et al., 2008; Magri, Vendramin, & Comps, 2006; Tzedakis, Emerson, & Hewitt, 2013; Willis, Rudner, & Sumegi, 2000).

In the traditional view, glacial refugia are known to be southern refugia for temperate species from all groups of organisms: the Iberian, Italian, Balkan peninsulas in Southern Europe (Hewitt, 1999, 2004; Taberlet, Fumagalli, Wust‐Saucy, & Cosson, 1998), where the influence of the glacial cycles was alleviated (Tzedakis, Lawson, Frogley, Hewitt, & Preece, 2002) and species could escape from cold dry climates and persist until they could repopulate Europe in the interglacials and after the last glacial. Nevertheless, recent studies suggested several additional refugia for temperate species beyond these peninsulas. These cryptic northern refugia are postulated for higher latitudes than the expected southern refugia (Bhagwat & Willis, 2008; Bylebyl, Poschlod, & Reisch, 2008; Magri et al., 2006; Willis & van Andel, 2004) and are defined as climatic islands with favorable conditions (Stewart & Lister, 2001), surrounded by unsuitable conditions. In the case of calcareous grasslands, this may have been rocky outcrops or steep sunny slopes with shallow dry soils (Ellenberg, 1988; Poschlod, Baumann, & Karlik, 2009) in deeply incised valleys providing microclimates for temperate species, allowing to survive where they normally would have perished (Flojgaard, Normand, Skov, & Svenning, 2009; Stewart & Lister, 2001). As soon as climate became warmer in the postglacial, recolonization of the surrounding steppe–tundra vegetation may have started from there. In this context, it must be mentioned that the following reforestation might have been held back before the Neolithic (see Bush, 1988), either by human with fire or by megaherbivores enlarging the potential habitats of calcareous grassland species besides naturally treeless sites like cliffs (Pokorný, Chytrý, & Juřičková, 2015; Svenning, 2002). Consequently, both wild animals and humans with domesticated animals may have contributed to species’ ranges and contributions.

Stewart, Lister, Barnes, and Dalen (2010) postulated a longitudinal oceanic‐continental gradient that is often ignored when speculating about recolonization of species along the latitudinal axis. The longitudinal gradient explains the expansion of steppe species and their inclusion in the Late Pleistocene ‘steppe–tundra’ biome. Accordingly, the occurrence of current postglacial steppe species is limited to eastern continental interglacial refugia which are determined by the longitudinal gradient. Occurrences in the west could therefore be interpreted as cryptic refugia (compare Kunes et al., 2008; Schmitt & Varga, 2012). On the other hand, there should be counterpart examples for oceanic species, because extension of arid climates during the late Pleistocene would have been an impediment to some taxa likewise cold climates. The ranges of oceanic species would have expanded during the moister interglacials and contracted to Western Europe during the glacial periods. Together with the latitudinal gradient, both work in tandem in defining suitable habitats of a species (Stewart et al., 2010).

In order to gain insight in the origin of a typical calcareous grassland species with submediterranean and oceanic requirements, we chose *Hippocrepis comosa* L. (horseshoe vetch). It has already been demonstrated that human activities have contributed at least since the early Neolithic to the migration of crops, weeds, and animals (Beebee & Rowe, 2000; Fjellheim, Rognli, Fosnes, & Brochmann, 2006; Poschlod & Bonn, 1998; Rosch, 1998; Willerding, 1986). It seems therefore quite possible that the migration of *H. comosa* is also related to human migration processes. The occurrence of *H. comosa* was first time documented for the Roman age in the lower Rhine Valley (Knörzer, 1996). Therefore, the question is whether or not the species came to Central Europe via Roman settlers. As Mediterranean species, there is also the possibility of spreading from Iberian or Balkan Peninsula. Exemplarily Poschlod (2014) claims the migration of dry grassland species from the Eastern Mediterranean region or southeast Europe through the migration of the first farmers of the linear ware ceramic culture (LBK) to Central Europe or from Western Europe through the La Hoguette culture.

Considering the climatic conditions in Central Europe during the Pleistocene, we postulate that *H. comosa* shifted westwards in the glacial periods due to the lateral expansion of continental climate and additional to its submediterranean character also southwards. We assume that *H. comosa* survived glaciations in western or southern refugia, but it cannot fully be excluded that the species also occurred in cryptic refugia in Central Europe.

Our aim was to identify glacial refugia and postglacial immigration routes of *H. comosa* to Central Europe, and we applied, therefore, two scientific approaches. Firstly, we used species distribution modeling (SDM) to predict suitable refugia during the Pleistocene with climate data. Within MaxEnt, a machine‐learning application, we initially calibrated a model containing actual distribution data of *H. comosa* in combination with a set of today's climate parameters (Elith & Leathwick, 2009). This model was then used to process climate data prevailing during the last glacial maximum to predict suitable refugia. Secondly, we applied amplified fragment length polymorphisms (AFLPs) as molecular markers to analyze the genetic variation within and among 38 populations of *H. comosa* from the whole distribution range to gain information about glacial refugia and recolonization routes of the species. More specifically we asked the following questions: (i) Which refugial areas served as source for the postglacial immigration of *H. comosa* to Central Europe? (ii) Where were the main migration routes from the refugia to Central Europe? (iii) Is there evidence for the long‐term survival of *H. comosa* in cryptic northern refugia?

## 2 Materials and Methods

### 2.1 Study species

For this study, we selected *Hippocrepis comosa* (horseshoe vetch), which is a typical calcareous grassland species with submediterranean and oceanic requirements. As mentioned by Schmidt et al. (2007), the species occurs primary in natural habitats and secondary in seminatural habitats. It also occurs in recent and ancient grasslands (Karlik & Poschlod, 2009) and seed exchange by grazing was shown possible (Müller‐Schneider, 1938). The native range of *H. comosa* covers middle and south European dry or semidry basiphilous and calciphilous grasslands or rocky cliffs (Brometalia erecti) and spring heath—Pine woods (Ericio‐Pinetum) or alpine calcareous grasslands (Seslerietalia albicantis).

### 2.2 Species distribution modeling

Information containing georeferenced occurrences of *H. comosa* was downloaded from the Global Biodiversity Information Facility (GBIF). The total number of downloaded data was 17,934 with about 7,000 locations clustered in the northern half of France. Therefore and because of the fact that the data set showed a mixture of grid based data (mainly in Germany, France, Spain, and UK) and pinpoint occurrences, an uniform raster was created with a point distance of 2.5 min in an unprojected coordinate reference system (WGS84) encompassing the total distribution area of the *H. comosa*. With this approach, sampling bias can be avoided (Wisz, Hijmans, & Li, 2008). The new distribution map was reduced to 2,794 points, 38 of them were additionally added from another study focusing on the same species (unpublished data). Geological parameters had to be excluded from our survey, as due to our grid based approach, the occurrence data of *H. comosa* would have been linked to incorrect edaphic values. Furthermore, to our knowledge, geological maps that describe the edaphic conditions during the LGM, especially in regard to current undersea areas, are not available.

To describe the climatic circumstances of the present age and the LGM (about 22,000 years ago), we used 19 bioclimatic variables (listed in Table A1, 1). The variables are derived from monthly mean temperature and precipitation and represent climatic annual trends, seasonality, and extreme conditions. Provided as separate climate layers at WorldClim (http://worldclim.org, Version 1.4, release 3, Hijmans, Cameron, Parra, Jones, & Jarvis, 2005), data were downloaded as grid (raster) format. The resolution of the data was 2.5 min (WGS84, unprojected). The current conditions involve interpolation of observed data from 1950 to 2000. As climatic data for the last glacial maximum conditions, two different reconstructions were used: CCSM4 and MPI‐ESM‐P. All gathered from WorldClim the original data were provided by the Coupled Model Intercomparison Project (CMIP5). The resolution was 2.5 min (WGS84, unprojected). As *H. comosa* exclusively occurs in Europe, geographic data were reduced to the European region. To avoid geographic bias, data were projected to an equal area projection (Europe Albers EAC). All GIS‐related work was carried out in ArcGis 10.2.2 (ESRI, Redlands, CA, USA).

Ecological niche modeling and the subsequent creation of the geographic distribution maps of *H. comosa* at present and past time was computed with the program Maxent, version 3.3.3 (Phillips & Dudik, 2008). The Maxent software uses a maximum entropy algorithm which is well suited for species habitat modeling using presence‐only data (Elith, Graham, & Anderson, 2006). Therefore, it is a proper method for predicting species distributions for both past‐ and future‐orientated scenarios (Hijmans & Graham, 2006). In a first step, we calibrated the model with the actual occurrence data of *H. comosa* together with all current 19 bioclimatic variables (Table A1, 1). The resulting potential species distribution model was then projected onto the climate conditions prevailing during the last glacial maximum. To validate the informative value of the model regarding species distribution, we used the area under the receiver operating characteristic (ROC) curve (AUC) (Fielding & Bell, 1997), which is an implemented validation routine within Maxent. The occurrence data were randomly partitioned into two groups: One group containing 75% of the data was used for the model calibration; the remaining 25% were used for model testing (Phillips, Anderson, & Schapire, 2006). High AUC values (>0.7) indicate a good model performance (Fielding & Bell, 1997). With following exceptions, we used the default settings in Maxent (Phillips & Dudik, 2008). The convergence threshold was set to 10^−5^, the maximum number of iterations was 5,000, and 15 replicates with the replicated run type “subsample” were made. The selection of the relevant climate data was automated. As threshold rule, we chose maximum test sensitivity and specificity (MTSS) to optimize the correct discrimination of presences and pseudoabsences in the test data (Hernandez, Graham, Master, & Albert, 2006; Jimenez‐Valverde & Lobo, 2007). The continuous logistic output of Maxent was transformed in a binary presence–absence map. The threshold value for presence was based on MTSS values which were averaged over 15 runs.

### 2.3 AFLP analysis

For the molecular analysis, plant material of *H. comosa* was sampled throughout the whole species range distribution on the European continent and embraced in total 588 individuals from 38 populations (Table 1). Each sample encompassed multiple fresh and healthy leaves which were dried in silica gel.

**Table 1:** Summary of the locations of all sampled populations. Longitudinal and latitudinal coordinates are given as decimal coordinates (WGS84)

Pop ID	Latitude	Longitude	Country
1	−5.945	42.932	Spain
2	−4.414	43.398	Spain
3	−3.485	41.977	Spain
4	−3.404	42.796	Spain
5	0.613	42.096	Spain
6	−0.107	50.901	United Kingdom
7	−0.399	44.762	France
8	1.010	49.321	France
9	2.182	42.875	France
10	4.339	44.972	France
11	4.609	44.802	France
12	4.778	50.298	Belgium
13	7.427	47.409	Switzerland
14	8.882	45.961	Switzerland
15	7.385	45.636	Italy
16	7.750	45.423	Italy
17	8.749	44.511	Italy
18	9.527	44.256	Italy
19	10.763	43.745	Italy
20	10.791	45.556	Italy
21	10.836	46.237	Italy
22	12.274	43.117	Italy
23	12.337	43.795	Italy
24	13.021	42.956	Italy
25	13.238	42.745	Italy
26	9.191	51.479	Germany
27	10.143	50.226	Germany
28	10.251	47.288	Germany
29	10.252	47.375	Germany
30	10.415	49.524	Germany
31	11.686	48.951	Germany
32	11.720	51.216	Germany
33	13.119	48.651	Germany
34	13.882	45.100	Croatia
35	16.368	43.894	Croatia
36	13.999	45.724	Slovenia
37	18.945	47.467	Hungary
38	21.655	41.368	Rep. of Macedonia

Pop. ID., Population identifier; Latitude/Longitude, geographic position.

DNA extraction followed CTAP protocol from Rogers and Bendich (1994) adapted by Reisch (2007) using 15 mg of the dried leaf samples. DNA contents were photometrically determined and adjusted to 7.8 ng DNA per 1 μl H_2_O. We chose the dominant marker analysis of amplified fragment length polymorphisms (AFLP, Vos, Hogers, & Bleeker, 1995; Zabeau & Vos, 1993) to produce loci over the whole genome (standardized AFLP protocol from Beckmann Coulter (Brea, USA)). Molecular analyses were conducted as described previously (Bylebyl et al., 2008). For the selective DNA amplification, we chose three pairs of primer (D2: GATGAGTCCTGAGTAACTA‐GACTGCGTACCAATTCAAC, D3: GATGAGTCCTGAGTAACAC‐GACTGCGTACCAATTCAGG, and D4: GATGAGTCCTGAGTAACAC‐GACTGCGTACCAATTCACA). PCR products were separated using capillary electrophoreses (CEQ 8000, Beckmann Coulter, USA). Data were exported as curve‐files and manually analyzed for the occurrence of strong, well‐defined fragments in Bionumerics 6.6 (Applied Maths, Kortrijk, Belgium). The presence or absence of fragments was transformed into a binary (1‐0) matrix, which served as basis for all further analysis. Individuals showing no clear banding signals were repeated or ultimately excluded.

### 2.4 Statistical analysis of the AFLP data

Based on the allele frequencies from the 0/1 matrix Nei′s Gene Diversity (Nei, 1972), Shannon′s Information Index (Shannon 1948), the number and percentage of polymorphic loci were calculated for each population using POPGENE v. 1.31 (Yeh, Yang, & Boyle, 1999). In order to make the results of Nei's gene diversity more comparable, an additional calculation was conducted at which the number of tested individuals was set to 12 for each population (lowest available amount). The 12 samples were chosen randomly with 50,000 iterations, and a mean value was calculated. The results were plotted onto the geographic coordinates of the sample locations. A base map provided by “Natural Earth” served as background for this and all following maps.

As an additional measure of divergence, the rarity of markers was calculated by frequency‐down‐weighted marker values (DW) (Schönswetter & Tribsch, 2005). The calculation of the DW values was performed via the r‐script AFLPdat (Ehrich, 2006). To grant equal sample sizes, for each population, 12 individuals were randomly selected with 10 iterations and a mean value of DW was calculated. The results were plotted onto the geographic coordinates of the sample locations. The value of DW is expected to be high in long‐term isolated populations where rare markers should accumulate due to mutations whereas newly established populations are expected to exhibit low values, thus helping in distinguishing old vicariance from recent dispersal (Schönswetter & Tribsch, 2005). We calculated a Pearson correlation coefficient for Nei′s Gene Diversity and DW value.

An analysis of molecular variance (AMOVA, Excoffier, Smouse, & Quattro, 1992) should give information about the genetic variance within and between populations. The two‐level AMOVA was performed within the program GENALEX v6.5 (Peakall & Smouse, 2012) and included 588 individuals of all 38 populations. Based on Euclidean pairwise genetic distances, the sums of squares were calculated (SSWP) and divided by the degrees of freedom (SSWP/*n* − 1). The resulting AMOVA‐SS diversity values per sample location were also presented cartographically. Permutation tests (9,999 iterations) were conducted to show significance.

The genetic structure and group assignment of the populations was investigated with Bayesian clustering in STRUCTURE v 2.3 (Pritchard, Wen, & Falush, 2009; Pritchard, Stephens, & Donnelly, 2000). The program performs a Markov chain Monte Carlo (MCMC) algorithm to assign the tested individuals into *k* groups based only on its genetic data, and not on population affiliation. The program was run with following parameters: no admixture ancestry model, correlated allele frequency model, *k* from 2 to 40, a burn‐in period of 10,000 followed by 10,000 iterations, 10 replicate runs. The most likely number of groups in the data set was determined via the calculation of Δk following the method of Evanno, Regnaut, and Goudet (2005). The results were plotted onto the geographic coordinates of the sample locations.

To identify spatial genetic patterns within the data set, a multivariate approach was conducted using spatial principal component analysis (sPCA). sPCA was carried out in R (Development Core Team, 2014) using package adegenet (Jombart, Devillard, Dufour, & Pontier, 2008). For this analysis, all 588 individuals of 38 populations were used. The populations’ geographic coordinates (WGS 1984) were projected to ETRS 1989 LCC. To avoid same coordinates of individuals in the same population, the coordinates were shifted randomly by a factor of 0.5. Unlike the analysis with STRUCTURE, the data for a sPCA do not have to meet Hardy–Weinberg expectations or linkage equilibrium. For the method, two matrices are necessary. The first one contains the relative allele frequencies of all individuals and the second embraces all spatial proximity information from the projected coordinates. The spatial proximity information matrix was gained from a connection network using Delaunay triangulation. Also, the second matrix was used to calculate a spatial autocorrelation using Moran's I (Moran, 1948). Moran's I ranges from +1 to −1, indicating a strong positive or negative spatial autocorrelation, respectively. In case of a positive spatial autocorrelation, a global structure in the data can be assumed. A Moran's I of zero indicates a totally random pattern. For a visual verification of the occurrence of spatial structures, a screeplot was drawn by plotting the variance of the sPCA against spatial autocorrelation (Moran's I). Supplementary, to statistically strengthen the previous visual findings, two Monte Carlo tests with 9,999 permutations each were conducted in order to detect global or local structures in the data set. As display, the genetic differentiation of the principal components was plotted onto the geographic coordinates.

## 3 Results

### 3.1 Species distribution modeling

**Figure A1:**
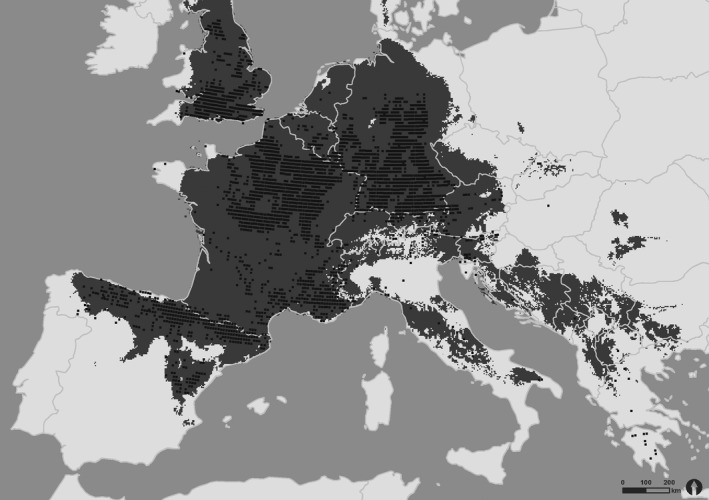
Distribution model of *H. comosa* in Europe based on current climate data. Dark gray areas indicate suitable habitats within the ecological niche; light gray area are unsuitable habitats for *H. comosa*. Black dots show the occurrence data gathered from GBIF and used for modeling. National boundaries are given

Species distribution modeling resulted in three maps, which show the predicted geographic distribution of *Hippocrepis comosa* for present time and the two different climatic assumptions (CCSM4 and MPI‐ESM‐P) for the LGM. The model for today's distribution of *H. comosa* displayed a good prediction of the reported locations (Figure A1, 1). Aberrations can be a result of an imprecise sampling design, due to the fact that first we had to rasterize all data and second an unsteady participation of European countries in providing occurrence data of the investigated plant species. Also, geological aspects were not included in the model. If taken into consideration, they would rule out areas with no occurrences of calcareous substrates like in Northern Germany and the Netherlands or silicate affected subsoils for example in France or the Czech Republic. Nevertheless, when the focus lies on climatic factors only, these regions provide suitable climatic habitats for *H. comosa*.

Of all 19 tested bioclimatic parameters, “Precipitation of Driest Quarter” (BIO17) had the highest influence on the prediction of suitable habitats of the actual occurrence of *H. comosa*, with a contribution to the model of 45%. Together with “Temperature Annual Range” (BIO7, percent contribution: 36%) and the “Isothermality” (BIO3, percent contribution: 8%, Isothermality = Mean Diurnal Range/Temperature Annual Range × 100) ranked second and third the first three parameters contribute with 89% to the final model.

**Figure 1:**
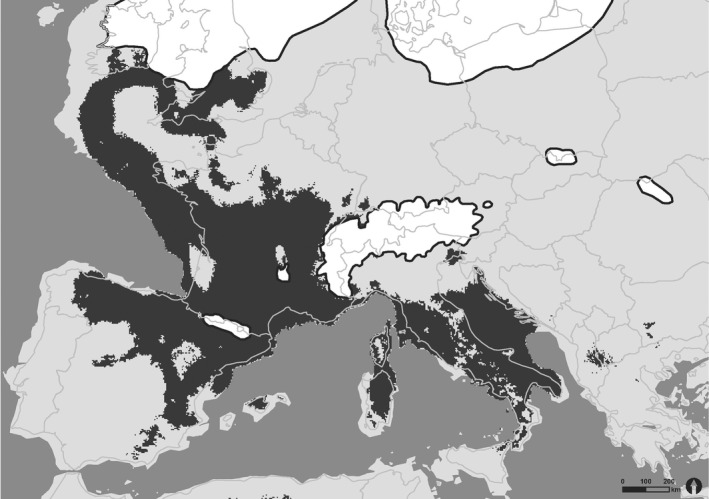
Species distribution model projection of *H. comosa* at the last glacial maximum (21,000 ya) based on the output of the MPI‐ESM‐P scenario. Dark gray areas indicate suitable habitats within the ecological niche; light gray area are unsuitable habitats for *H. comosa*. Ice shields are shown in white with a dark outline. National boundaries represent today's European land area

**Figure A2:**
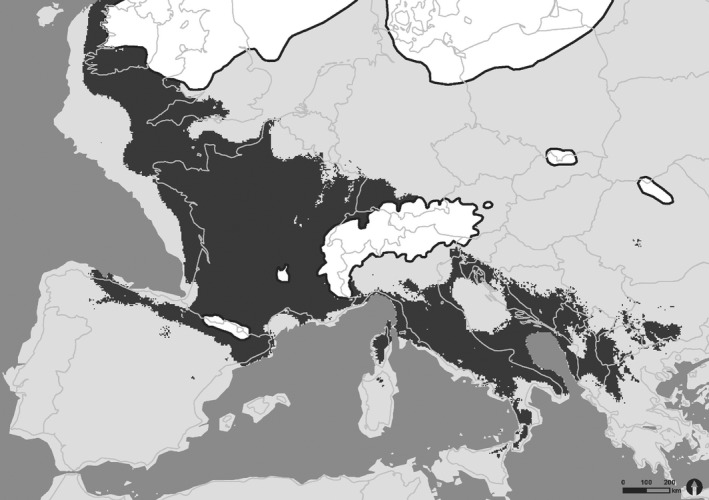
Species distribution model projection of *H. comosa* at the last glacial maximum based on the output of the CCSM4 scenario. Dark gray areas indicate suitable habitats within the ecological niche; light gray area are unsuitable habitats for *H. comosa*. Ice shields are shown in white with a dark outline. National boundaries represent today's European land area

Both models for the distribution of *H. comosa* at the LGM (about 22,000 years ago) effected good performances with AUCs of 0.890 for CCSM4 (Figure A2) and 0.889 for MPI‐ESM‐P (Figure 1). In both predictions of suitable climatic habitats for *H. comosa,* similar distribution maps were computed. Separated by the Alps two major clusters could be distinguished: One situated in the west of Europe involving today's submersed lands west of France and the UK, France, and the north of Spain and a second embracing Italy, the Adriatic Sea, and parts of the Balkan Peninsula. Corresponding to the postulated refugia on the Peninsulas of Iberia, Italy, and Balkan, these regions formed suitable habitats for *H. comosa* during the LGM. In general, the model based on MPI‐ESM‐P data showed a stronger tendency of shifting suitable habitats toward the south and west than the CCSM4 model.

### 3.2 AFLP analysis

**Figure 2:**
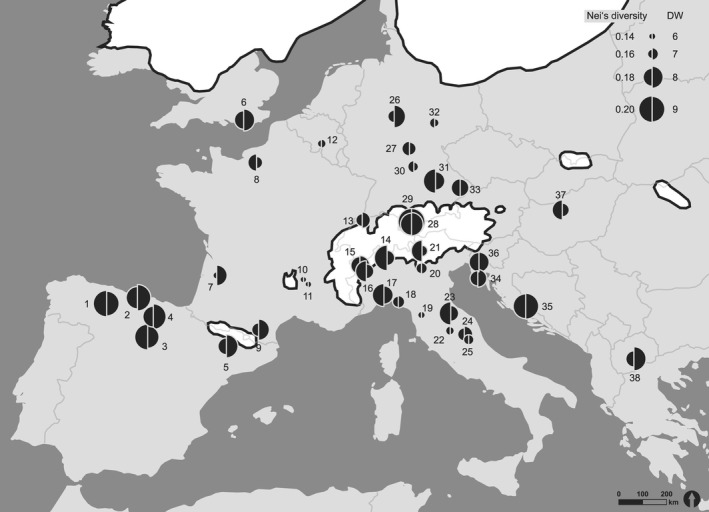
Map of Nei's gene diversity (left semicircle) and frequency‐down‐weighted marker values (DW, right semicircle) for each surveyed population. The different sizes of the circles indicate different absolute values. Ice shields are shown in white with a dark outline. National boundaries represent today's European land area

In the AFLP analysis of 588 individuals with three primer combinations, 271 unambiguous fragments were selected ranging between 60 and 420 bp and of which 98.16% were polymorphic (D2 CTA‐AAC: 111 fragments, D3 CAC‐AGG: 87 fragments, D4 CAC‐ACA: 73 fragments). The within‐population genetic variation was calculated as four different measures (Table 2). All genetic variation values were consistently lowest in population no. 11 in France and highest in population no. 29 in Germany. Mean percentage of polymorphic loci (% PPL) was 56.7%, ranging between 46.3 and 64.7. Mean Shannon's information index (SI) was 0.28, ranging between 0.21 and 0.32. Mean Nei's gene diversity yielded for all individuals was 0.18, ranging from 0.14 to 0.21. Mean Nei's gene diversity for 12 individuals was 0.18, ranging from 0.13 to 0.21. Higher values for genetic variation (He ≥ 0.19) were recorded only in southern populations, like the Iberian (He = 0.2 and 0.21), the Italian (He = 0.19), the Balkan Peninsula (He = 0.20), south of the Alps (population no. 14, He = 0.2), except from one population in the northern Alps (population no. 29, He = 0.21), and north of the Alps in Germany (population no. 31). Lower values (He ≤ 0.16) only occurred in northern populations and on the Italian peninsula, but not in the two other peninsulas (Figure 2).

**Table 2:** Genetic variation within the populations of *H. comosa*

Pop. ID	CC	*N*	PL	PPL	He	He_12_	SI	DW_12_	SSWP/*n* − 1
1	ES	14	171	62.9	0.21	0.208	0.32	8.965	29.95
2	ES	16	165	60.7	0.20	0.189	0.30	10.453	28.28
3	ES	15	171	62.9	0.21	0.201	0.31	8.531	30.94
4	ES	16	159	58.5	0.19	0.183	0.29	9.131	27.27
5	ES	15	159	58.5	0.18	0.176	0.27	8.842	27.40
6	UK	15	152	55.9	0.19	0.182	0.28	8.055	25.79
7	FR	15	139	51.1	0.16	0.153	0.24	7.906	22.50
8	FR	16	150	55.2	0.18	0.176	0.28	6.981	26.68
9	FR	16	153	56.3	0.18	0.173	0.27	7.944	26.35
10	FR	16	134	49.3	0.14	0.137	0.22	4.831	20.96
11	FR	16	126	46.3	0.14	0.132	0.21	4.907	20.21
12	BE	15	133	48.9	0.16	0.154	0.24	6.727	22.56
13	CH	16	152	55.9	0.18	0.171	0.27	7.484	26.81
14	CH	15	164	60.3	0.21	0.200	0.31	7.337	28.66
15	IT	16	155	57.0	0.19	0.181	0.28	7.782	27.13
16	IT	16	160	58.8	0.19	0.186	0.29	7.297	27.28
17	IT	16	174	64.0	0.20	0.195	0.31	7.760	30.28
18	IT	16	144	52.9	0.17	0.164	0.26	7.046	23.66
19	IT	16	134	49.3	0.16	0.153	0.24	6.153	22.82
20	IT	16	138	50.7	0.17	0.164	0.25	6.853	24.88
21	IT	16	159	58.5	0.19	0.185	0.29	6.829	27.07
22	IT	16	140	51.5	0.17	0.163	0.25	6.590	22.90
23	IT	16	163	59.9	0.20	0.194	0.30	7.379	28.75
24	IT	16	148	54.4	0.18	0.171	0.26	7.566	27.50
25	IT	16	159	58.5	0.17	0.163	0.26	6.762	26.05
26	DE	16	143	52.6	0.18	0.171	0.27	8.361	26.11
27	DE	16	156	57.4	0.18	0.174	0.27	7.096	27.99
28	DE	15	162	59.6	0.20	0.189	0.29	8.835	28.42
29	DE	16	176	64.7	0.21	0.206	0.32	10.339	31.96
30	DE	16	146	53.7	0.17	0.167	0.26	6.743	25.19
31	DE	16	174	64.0	0.20	0.197	0.31	7.775	30.55
32	DE	16	159	58.5	0.17	0.163	0.26	6.665	26.10
33	DE	12	147	54.0	0.18	0.181	0.27	7.578	27.79
34	HR	15	159	58.5	0.18	0.174	0.27	7.663	26.16
35	HR	14	164	60.3	0.20	0.198	0.31	9.890	27.62
36	SL	15	165	60.7	0.19	0.182	0.29	7.880	27.90
37	HU	13	153	56.3	0.19	0.183	0.28	7.180	27.67
38	MK	16	154	56.6	0.18	0.175	0.27	8.888	25.96
	Mean	15.5	154	56.7	0.18 ± 0.01	0.177 ± 0.01	0.28 ± 0.02	7.658 ± 0.200	26.63 ± 0.43

Pop. ID., Population identifier; CC, country code; *N*, sample size; PL, number of polymorphic loci; PPL, percentage of polymorphic loci; He, Nei's gene diversity (with standard error); He_12_, Nei's gene diversity for 12 randomly chosen individuals (with standard error); SI, Shannon Index (with standard error), rarity value (DW_12_), AMOVA‐SS (SSWP/*n* − 1).

The survey of the rarity of markers revealed DW values ranging from 4.83 in an Italian population (no. 10) to 10.5 in a Spanish population (no. 2), with an average of 7.66 (SE = 0.2; see Table 2). The highest values were recorded either in populations of the Iberian Peninsula (values between 8.35 and 10.5), the Balkan Peninsula (values between 8.89 and 9.89), or in some northern populations, in Central Germany (DW = 10.3), Bavarian Alps (DW = 8.84 and 8.36), and the United Kingdom (DW = 8.05). Low values occurred in northern populations and on the Italian, but not on the Iberian and Balkan peninsulas (Figure 2). There were highly significant positive correlations between DW and all genetic variation measures (*r* = .729413, *p* < .0001, *t* = 6.3977), meaning that a high number of rare markers (high DW) were associated with high genetic variation.

**Figure 3:**
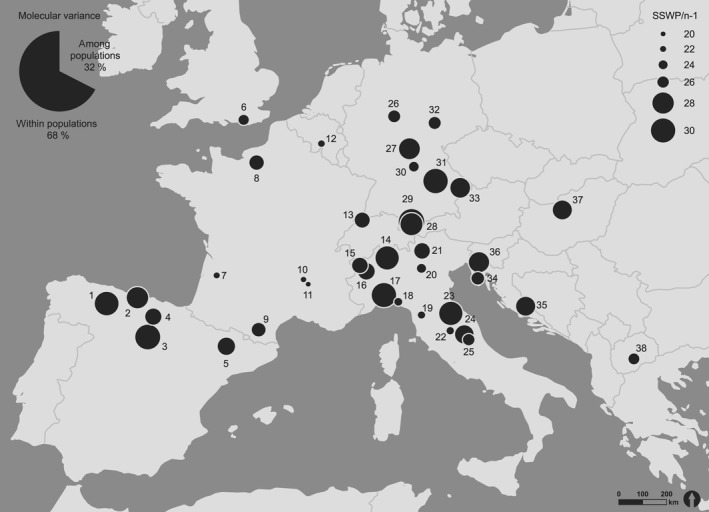
Map of AMOVA‐SS values for each surveyed population. The different sizes of the circles indicate different absolute values of molecular variance

The analysis of molecular variance (AMOVA) involving all populations without classification of regions revealed a total molecular variance within the populations of 68%. This leaves a strong differentiation among the populations with a molecular variance of 32% (Figure 3). The results were highly significant (*p* < .001). All AMOVA‐SS values are given in Table 2.

**Figure 4:**
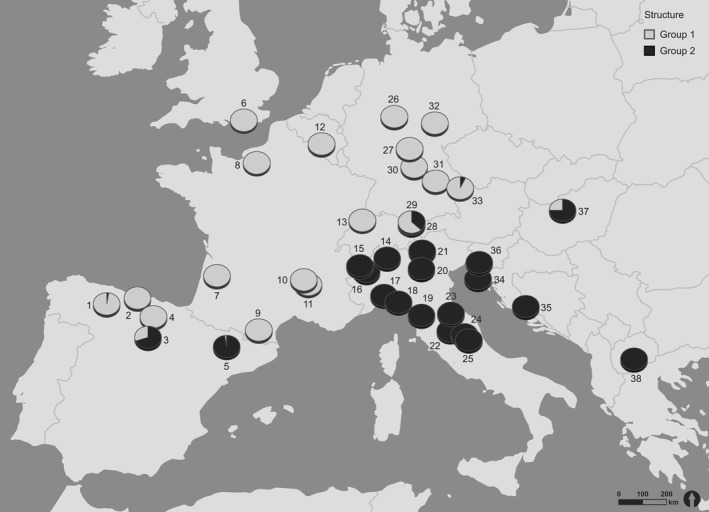
Results from STRUCTURE analysis. The surveyed populations of *Hippocrepis comosa* were plotted onto geographic coordinates of. As STRUCTURE proposed a two‐group solution, each population was assigned according its associated group

**Figure A3:**
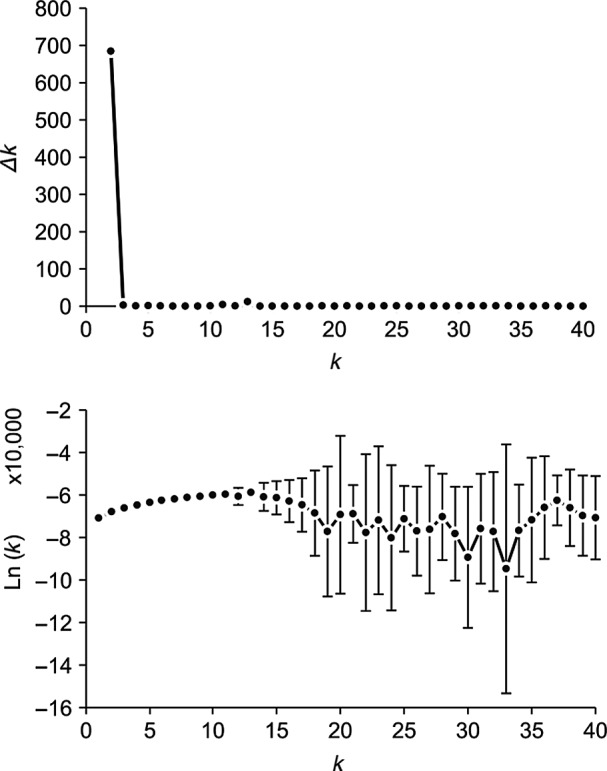
Analysis of the STRUCTURE runs. Relationship between the number of proposed groups k, Δk and Ln(k), respectively. The most likely number of groups with highest Δk=684.8 was found with k=2

The result of the Bayesian clustering conducted with STRUCTURE supports a clear assignment of the individuals into two groups (*k* = 2, Figure A3). Besides this most likely number of clusters, a very small probability for 13 groups was found, one group involving populations of Western and Central Europe (Spain, France, United Kingdom, Belgium, Switzerland, Germany), and a south Eastern European group (Italy, Slovenia, Croatia, Macedonia). Only at the boundary points of the two groups in Spain (no. 3 and 5), Germany (no. 28, 29, and 33), and Hungary (no. 37), the 100% assignment into one of the groups was diluted, resulting in some admixed populations (Figure 4).

**Figure A4:**
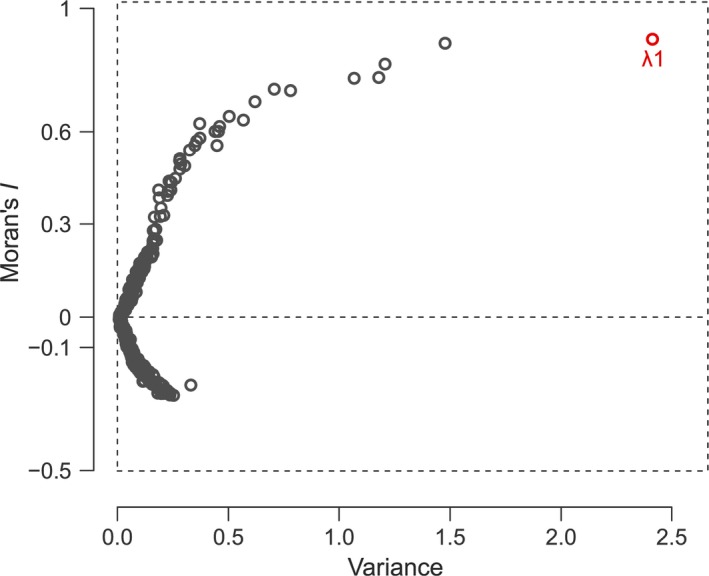
Screeplot of the total data set. The eigenvalues of the sPCA are arranged by its variance (*x*‐axis) and Moran's I (spatial autocorrelation, *y*‐axis). As the *y*‐axis represents Moran's I, positive scores indicate global structures. Moran's I shows a high‐spatial autocorrelation (>0.8). Due to its largest positive eigenvalues λ1, the first global score can be clearly distinguished from all other values and therefore was chosen for further interpretation. The maximum attainable variance from an ordinary PCA is shown as the dashed vertical line on the right side of the graph. The range of variation of Moran's I is limited by the horizontal dashed lines

Likewise, the results of the sPCA in the assignment of the populations into two groups revealed a similar pattern. All 588 individuals were included in the spatial analysis. The spatial PCA was based on Delaunay triangulation as connection network. The eigenvalues of the sPCA in dependence of its Moran's I and variance are shown in a screeplot (Figure A4). The eigenvalue of the first global score λ1 could clearly be distinguished from all other eigenvalues due to its higher levels of variance and spatial autocorrelation. This indicates the existence of spatial structures in the data, which was subsequently tested with global and local Monte Carlo tests (9,999 iterations). As the global test showed a significant (*p* < .0001) result, and with the screeplot in mind, a global spatial structure was assumed for the data set. The local Monte Carlo test was not significant.

**Figure 5:**
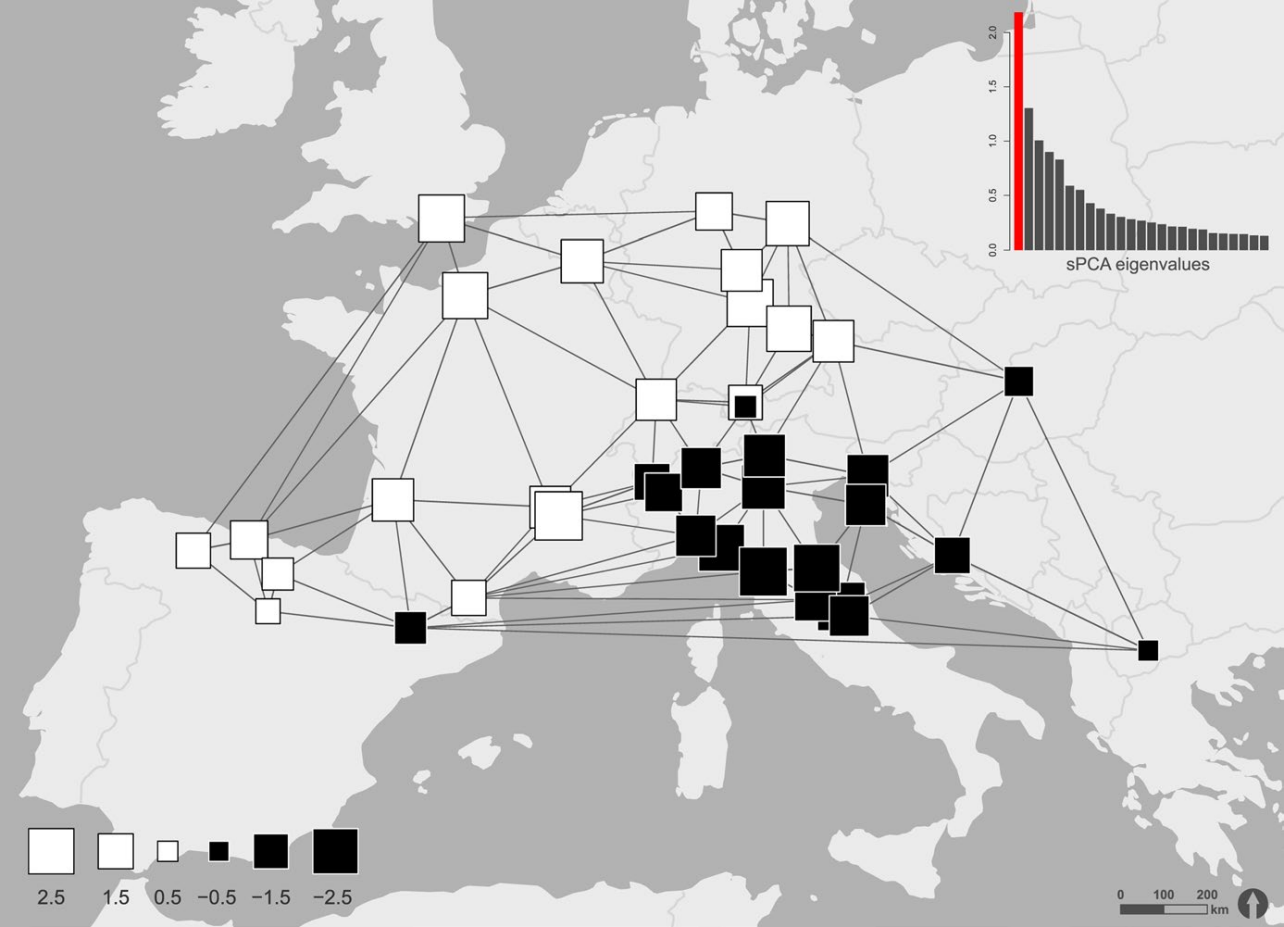
Graphical display of the spatial distribution of all surveyed populations with the values of the first positive (global) sPCA score. The different sizes of the squares indicate different absolute values. Large black squares are well differentiated from large white ones, while small squares show less differentiation. On the map, the genotypes differentiate in two distinct clusters, one in the in the northwest and one in the southeast. The used connection network based on Delaunay triangulation is shown with gray lines. On the top right position of the map, the first 25 sPCA‐positive scores are shown

Figure 5 shows the eigenvalues of the first global score plotted against geographic coordinates. Black squares indicate positive and white squares negative values of the scores. The size of the squares represents different absolute values. Therefore, large‐sized squares from both colors are highly differentiated, while small‐sized squares indicate only small differentiation. A clear distinction between two clusters can be drawn, one involving populations of Western and Central Europe (Spain, France, United Kingdom, Belgium, Switzerland, Germany) and the other one including populations from south Eastern Europe (Italy, Hungary, Slovenia, Croatia, Macedonia). A Monte Carlo Mantel test (10,000 iterations) for a correlation between geographic and genetic distances was highly significant (*p* < .0001).

## 4 Discussion

### 4.1 Species distribution modeling

As the predicted present‐day distribution of *H. comosa* showed a good match with the actual distribution, we considered the following SDMs predictions suitable habitats during the last glacial maximum as convincing. Our findings support the generally accepted assumption of northern temperate species outlasting the LGM in south located European refugia (Bennett, Tzedakis, & Willis, 1991; Hewitt, 1999, 2000; Huntley & Birks, 1983; Taberlet et al., 1998). Additionally, results of the species distribution models suggest the existence of possible refugia in France and along the Atlantic coast up to the UK. Both models (CCSM4 and MPI‐ESM‐P) show differences in this area. While CCSM4 predicts suitable habitats for almost entire France and even parts of southwest Germany, the MPI‐ESM‐P model draws the restriction further west. The reason lies most probably in the fact that both models make different assumptions regarding annual precipitation. While CCSM4 model only shows drier summer in Central Spain and Northern Italy but not in Central Europe, the opposite conditions are predicted by the MPI‐ESM‐P model (PMIP3, Braconnot, Harrison, & Kageyama, 2012). Given that the main contributing parameter in our models was “Precipitation of Driest Quarter”, this assumption seems reasonable. Nevertheless, both SDM predicted vast areas of nowadays submerged land as suitable habitats for *H. comosa* as a species adjusted to oceanic climate. The up to 110 m lower sea level (Ruddiman & Thomson, 2001) revealed ice‐free land west of France and the UK of several 100 km wide. Beyond climatic parameters, it is uncertain whether this land masses provided the proper calcareous substrates for *H. comosa* and therefore could have served as refugia. Otherwise several studies including flora and fauna (Boston, Montgomery, Hynes, & Prodohl, 2015; Ohlemüller, Huntley, Normand, & Svenning, 2012; Svenning, Normand, & Kageyama, 2008) are supporting the existence of these north western potential refugia.

### 4.2 Eastern and western lineages and postglacial migration

AFLP analysis is a powerful tool in revealing glacial refugia and postglacial immigration processes, as we show here on an example of the calcareous grassland species *H. comosa*. Based upon our results, we assume that *H. comosa* followed a contraction–expansion model, whereby the species was restricted to traditional southern refugia during the LGM following a latitudinal temperature and a longitudinal humidity gradient and expanded into the rest of Europe afterward. Our results indicate additional cryptic refugia at the western shores of France and UK.

Both spatial analyses (STRUCTURE and sPCA) identified two almost completely distinct lineages of *H. comosa*: one group involving populations of the Iberian peninsula and Western Europe (Spain, France, United Kingdom, Belgium, Switzerland, Germany) and the other including populations from the Italian and the Balkan peninsulas (Italy, Croatia, The Former Yugoslav Republic of Macedonia, Slovenia). Only a few admixed populations were detected, located in Hungary, Germany, and Spain. According to these spatial results, two traditional southern refugia (Hewitt, 1999, 2004; Taberlet et al., 1998) could be confirmed: Iberia as a southwestern refugia and Italy and the Balkans as southeastern refugia, enclosing the admixed populations in Spain, South Germany, and Hungary as contact zones. Contact zones have been reported to cluster in the Alps, Central Europe, northern Balkans, and the Pyrenees (Hewitt, 2000; Taberlet et al., 1998), resulting in an accumulation of genotypes from both groups. Therefore, we assume that *H. comosa* repopulated Central Europe from Iberia (Pyrenees) to France, Britain, Belgium, Switzerland, and Germany until its eastern border, where the populations admixed with populations that were migrating from Italy and the Balkans up north. It has been shown before that in contrast to Iberian lineages, Italian genomes rarely populated Northern Europe, as the ice‐capped Alps prevented their northward expansion (Hewitt, 2000; Taberlet et al., 1998). This barrier is regarded as explanation of the relatively low species’ and genetic diversity of northern populations compared to southern populations (Hewitt, 2000). Considering the values for Nei's gene diversity and the rarity index (DW)‐values, *H. comosa* populations of the Balkan Peninsula may have served as refugium from where the Italian populations were founded subsequently.

### 4.3 Southern and cryptic northern refugia

The most important refugial areas are geographic regions where species persisted throughout several full glacial/interglacial cycles (each 100–120 kyr in duration). These so‐called true refugia (Stewart & Dalen, 2008) are expected to possess higher genetic variability compared with surrounding recolonized regions (Comes & Kadereit, 1998; Taberlet et al., 1998; Tzedakis et al., 2013). In contrast, recent dispersal might lead to genetic depauperation due to founder effects. Supporting this theory, the highest values for genetic variation of *H. comosa* were recorded almost entirely in southern populations, like the Iberian, the Italian, the Balkan Peninsula, and south of the Alps. Other above‐average genetically diverse populations were located in the Alps and in Germany. The former can be ascribed to the above‐mentioned hybridization of the western and the southeastern lineage (Petit, Aguinagalde, & de Beaulieu, 2003; Provan & Bennett, 2008; Tzedakis et al., 2013). We found different explanations for the high genetic variation of the latter. Firstly, it may be the result of recent genetic exchange due to grazing management, which is more prevalent in the area of the Jurassic mountains in Germany than in the northern German populations. Paun, Schonswetter, Winkler, Tribsch, and Consortium (2008) criticized the use of genetic variation for identification of refugia as they may in fact reflect current processes (genetic exchange and population sizes) instead of historical processes. Secondly, the high genetic variations may also have resulted from a phalanx way of recolonization from south to north or can be interpreted as a legacy from Younger Dryas cold reversal (Hewitt, 1999, 2000, 2004). A reduction of northern populations during this cold period could have led to high diversity populations due to the mixture with recolonizing linages during the Holocene (Tzedakis et al., 2013). In order to circumvent these confusions, a second parameter, the rarity index (DW) was used, as it is a better indicator of historical processes (Paun et al., 2008). The value of DW is expected to be high in long‐term isolated populations where rare fragments could accumulate due to mutations, whereas young populations are expected to show low values, thus helping in distinguishing old vicariance from recent dispersal. Refugial populations and recolonized regions would on the one hand contain identical fragments but due to drift rare fragments would accumulate and distinguish them from other refugial areas and surrounding younger populations that would be less divergent (Provan & Bennett, 2008; Schönswetter & Tribsch, 2005; Tzedakis et al., 2013). According to this, we identified additional northern populations of *H. comosa* that are located far from the traditional southern refugia which possessed high DW values, supposing cryptic refugia in UK, the Alps, and Central Germany. Such cryptic refugia have previously also been identified for other grassland species like *Bromus erectus* (Sutkowska, Pasierbinski, Warzecha, Mandal, & Mitka, 2013) or grassland related pine forest species like *Polygala chamaebuxus*. Although it must not be ignored that the rarity index might be overestimated, as related southern populations with these alleles may not have been investigated or distinct gene patches might result by gene surfing on the leading edge (Tzedakis et al., 2013). Nevertheless, we found a significant correlation of the rarity index (DW) and Nei's gene diversity (He), which means that in our study, rare fragments accumulation and high genetic diversity came along with each other, pointing toward “true refugia”. The German population with a very high rarity index and relatively low genetic variation may indicate an isolated refugium during the LGM, similar to alpine populations of *Ranunculus glacialis* (Paun et al., 2008). These results also coincide with Dengler, Janisova, Torok, and Wellstein (2014), who proposed a continuous existence of palearctic grassland at least since the Pleistocene (2,400 ka). During glaciations, grasslands covered most of the continent as steppe–tundra over permafrost and as xerothermic grassland further in the south. During the interglacials (Lang, 1994; Pärtel, Bruun, & Sammul, 2005), grasslands were mostly replaced by forests, apart from small‐scale areas on soils that impede tree growth (drought, shallow ground, instability (Ellenberg & Leuschner, 2010; Janišová, Bartha, Kiehl, & Dengler, 2011; Karlik & Poschlod, 2009) and reoccurring events like fire, wind throw (Hejcman, Hejcmanova, Pavlu, & Benes, 2013), grazing by wild herbivores (Vera, 2000), or human activity since the Mesolithic (Bush, 1988; Simmons & Innes, 1981). As *H. comosa* was described frost tolerant by Hennenberg and Bruelheide (2003) and it is growing up to 2000 m a.s.l. in the Alps, it seems reasonable to assume that the species might have occurred in Central Europe during the LGM on outcrops under favorable conditions.

### 4.4 Latitudinal and longitudinal constraints of its distribution

On a global scale, it applies for past, present, and future that wild species ranges are primarily determined by climate (Normand, Ricklefs, & Skov, 2011; Pearson & Dawson, 2003; Willis & Whittaker, 2002). Besides, there are additional parameters influencing the accessibility of species like life form, dispersal ability and LGM refugia location (glacial contraction), generation time, habitat adaptation, competition with established vegetation, soil development, geographic barriers and human habitat fragmentation (Normand et al., 2011), which may have resulted in a current disequilibrium (postglacial colonization) of species ranges within the actual climate (migrational lag). Regarding the distribution of *H. comosa* in Europe, we assume that it has fully expanded to its potential range. The northern distribution limitation as it is today was investigated on a regional scale by Hennenberg and Bruelheide (2003), showing a reduced fitness (reduced seed setting), which correlated with effective air temperature measured at a height of 10 cm above ground. The area of climatically suitable habitats was also shown in the present time species distribution model for this species. In addition, soil composition impedes an expansion further north and east than its present status (Hartmann & Moosdorf 2012). Therefore, we depicted that the current occurrence of *H. comosa* is mainly limited due to climate and soil factors and that the distribution pattern is not limited by reduced accessibility.

The declining temperature at the beginning of the last ice age was with no doubt the main driving factor influencing migration of plants following a latitude gradient to their southern refugia (Taberlet et al., 1998). Nevertheless, likewise Stewart et al. (2010), we emphasize that not only this latitudinal gradient should be taken into consideration. The longitudinal gradient representing an increasing continental climate from west to east might have had an impact on the migration to suitable habitats especially for species classified as oceanic like *H. comosa*. Due to declining precipitations in combination with a lower sea level, which comprised great land masses between England and Europe, the oceanity of Central Europe decreased. Regarding the migration of plants at that time, it is our opinion that oceanic plants were not only forced to move southwards but also westwards to maintain their climatic niche. Furthermore, the Alps and the Carpathian Mountains have acted as natural barrier hindering exchange and migration of plants toward Italy. The north and Central European populations of *H. comosa* therefore might have followed a combination of both vectors to the south and the west pointing toward a potential refugium in Spain, which can be an explanation for our cluster including Spain, France, Belgium, UK, Switzerland, and Germany. After the LGM, *H. comosa* migrated to Central Europe, but no further east because of the climatic limitations and edaphic barriers. Nevertheless, following the results of the species distribution modeling describing climatic niches during the LGM, there is also a possibility of cryptic northern refugia located in the UK and France, which could have served as additional places of origin for a recolonization.

For a successful recolonization following the LGM, *H. comosa* could rely on at least two mechanisms of seed dispersal. Endozoochory via herbivores (Fischer, Poschlod, & Beinlich, 1996; Müller‐Schneider, 1938; von Oheimb, Schmidt, Kriebitzsch, & Ellenberg, 2005) and epizoochory, implying a transportation with soil material in the hooves, which was reported for sheep and cattle (Fischer et al., 1996; Poschlod & Bonn, 1998) and affects the genetic structure of plant populations (Willerding & Poschlod, 2002). However, this way of transport can most probably also be transferred to other hoofed animals like red or roe deer. Therefore, we would designate *H. comosa* as a species with a high long‐distance dispersal potential, especially because of the fact, that hoofed animals can bridge distances of several kilometers per day (Pépin, Adrados, Mann, & Janeau, 2004). This becomes obvious when considering the work of Skog, Zachos, and Rueness (2009) and Meiri, Lister, and Higham (2013), presenting the recolonization and phylogeography of European red deer (*Cervus elaphus*). Based on modern and ancient DNA, this study strikingly resembles our findings for *H. comosa*, showing a restriction of *Cervus elaphus* to southern refugia during the LGM and a recolonization of Western and Northern Europe originating from Iberia. This very similar phylogeographic pattern may lead to the assumption that *H. comosa* expanded to Central Europe via *C. elaphus* or other equivalent herbivores (wild horse, roe deer; Pakeman, 2001).

Whether and to what extent human activities may have contributed to the expansion of *H. comosa* because the late stages of the Upper Paleolithic cannot be answered. But grasslands may have continuously existed since the LGM (Bush & Flenley, 1987; Bush, 1988; Pokorný et al., 2015), also because of human practices (Poschlod, 2014). The La Hoguette culture (~7,500 y.a.) showed first steps toward nomadic goat and sheep breeding (Gronenborn, 2003) and therefore could have directly influenced the dispersal of *H. comosa* via epizoochory (Müller‐Schneider, 1938) from Southern France to Germany. This assumption demonstrates that a more interdisciplinary approach to this subject is necessary in order to fully understand the recolonization of Central Europe by plants, animals, or humans. A strengthened cooperation of phylogenetics and archeology could deliver intriguing new insights in the genesis of our environment.

## 5 Conclusions

Based on the present study on the previous and current distribution of *H. comosa*, we could demonstrate that a comprehensive climatic approach including a second driving factor can lead to a better understanding of historical and present developments. The traditional latitudinal temperature gradient as major parameter was extended by a longitudinal humidity gradient which both work in tandem defining the suitable habitats during the LGM. Of the two detected clearly distinguished phylogeographic clusters, one in Western Europe ranging from Spain to Germany and the other embracing parts of south Eastern Europe, only the western refugia contributed to the recolonization of Central Europe. As the climate became drier in Central Europe during the LGM, *H. comosa* evaded to moister climates, which prevailed in Western Europe. The results of our survey furthermore provide evidence for northern locations in France and the UK that could have served as cryptic refugia. For the postglacial recolonization, *H. comosa* could have benefited from habitats shapes by humans and zoochory, which provided a long ranged dispersal to Central Europe. Thus integrated approaches incorporating multidisciplinary knowledge might be the best way to approximate and illuminate historical and present processes.

## Conflict of Interest

The authors declare that they have no conflict of interest.

## Author Contributions

M.L. and S.T. collected the plant material and performed the analyses. M.L., S.T., P.P., and C.R. contributed to manuscript writing.

## Appendix 1

### 1.1

**Table A1:** List of all climatic variables used to predict the climatic conditions in this study. The grid resolution was 2.5 min (http://worldclim.org)

BIO10 = Annual Mean Temperature
BIO20 = Mean Diurnal Range (Mean of monthly (max temp − min temp))
BIO30 = Isothermality (BIO2/BIO7) (* 100)
BIO40 = Temperature Seasonality (standard deviation *100)
BIO50 = Max Temperature of Warmest Month
BIO60 = Min Temperature of Coldest Month
BIO70 = Temperature Annual Range (BIO5‐BIO6)
BIO80 = Mean Temperature of Wettest Quarter
BIO90 = Mean Temperature of Driest Quarter
BIO10 = Mean Temperature of Warmest Quarter
BIO11 = Mean Temperature of Coldest Quarter
BIO12 = Annual Precipitation
BIO13 = Precipitation of Wettest Month
BIO14 = Precipitation of Driest Month
BIO15 = Precipitation Seasonality (Coefficient of Variation)
BIO16 = Precipitation of Wettest Quarter
BIO17 = Precipitation of Driest Quarter
BIO18 = Precipitation of Warmest Quarter
BIO19 = Precipitation of Coldest Quarter
